# Effects of α-Linolenic Acid on Seminiferous Tubule Morphology and UCHL1-Positive Cells in Cultured Testicular Tissue Fragments from a 3-Month-Old *Hu* Ram: A Pilot Study

**DOI:** 10.3390/ani16182873

**Published:** 2026-09-12

**Authors:** Rongyu Yao, Fadi Li, Xiuxiu Weng, Wanhong Li

**Affiliations:** State Key Laboratory of Herbage Improvement and Grassland Agro-Ecosystems, College of Pastoral Agriculture Science and Technology, Lanzhou University, Lanzhou 730020, China; 120220900691@lzu.edu.cn (R.Y.); lifd@lzu.edu.cn (F.L.); wengxx@lzu.edu.cn (X.W.)

**Keywords:** α-linolenic acid, testicular tissue fragments, *Hu* ram, seminiferous tubule morphology, transcriptomics

## Abstract

α-linolenic acid (ALA) has been reported to benefit testicular function. However, dietary ALA is extensively metabolized in the rumen, whereas isolated cell models do not retain seminiferous tubule structure or interactions among testicular cells. This pilot study examined the effects of ALA on seminiferous tubule morphology, UCHL1-positive cells, and gene expression in cultured sheep testicular tissue fragments. Tissue from one 3-month-old *Hu* ram (*n* = 1) was cultured for 50 d in control medium (CK) or medium containing 5 μM ALA. Three independently cultured fragments per condition were analyzed. Histology, UCHL1 immunofluorescence, RNA sequencing, and qRT-PCR were performed. ALA-treated fragments showed greater seminiferous tubule diameter, a lower proportion of empty space, and more UCHL1-positive cells per seminiferous tubule. Gene sets related to ATP-dependent chromatin remodeling, DNA replication, and male meiotic nuclear division were enriched in ALA-treated fragments, whereas immune gene sets were enriched in CK fragments. The relative expression levels of *SYCP3* and *MKI67* were higher in ALA-treated fragments than in CK fragments. Overall, these preliminary findings suggest that ALA treatment was associated with morphological and transcriptional changes in cultured sheep testicular tissue. Further studies using tissues from multiple rams are needed to confirm these findings.

## Simple Summary

Alpha-linolenic acid is an essential fatty acid that may benefit testicular function. In sheep, most dietary alpha-linolenic acid is metabolized during digestion before it reaches the testis. Studies of isolated cells cannot show how intact testicular tissue responds. This pilot study compared the structure of cultured sheep testicular tissue, the number of UCHL1-positive cells, and gene expression between control and alpha-linolenic acid-supplemented cultures. Testicular tissue fragments from one 3-month-old *Hu* ram were cultured for 50 days in control medium or medium supplemented with alpha-linolenic acid. Culture with alpha-linolenic acid was associated with greater seminiferous tubule diameter and a lower proportion of empty space within the tubules on days 30 and 50. The number of UCHL1-positive cells per seminiferous tubule was also higher on day 50. Gene expression analysis showed that gene sets related to immune and inflammatory responses were enriched in the control fragments, whereas gene sets related to DNA replication and germ cell processes were enriched in the alpha-linolenic acid-treated fragments. However, all tissue fragments were obtained from the same ram. Therefore, these findings are preliminary and require confirmation using tissues from multiple rams.

## 1. Introduction

α-linolenic acid (ALA) is an essential n-3 polyunsaturated fatty acid that cannot be synthesized by mammals and must be obtained from the diet [1]. It is widely found in plant sources, such as flaxseed, chia seeds, and walnuts. Sertoli and peritubular cells preferentially utilize ALA compared with other essential fatty acids [2]. In addition, flaxseed oil rich in ALA and fish oil rich in eicosapentaenoic acid (EPA) and docosahexaenoic acid (DHA) had different effects on bull sperm, with higher sperm motility after flaxseed oil supplementation [3]. These findings suggest that ALA may have a specific biological role in male reproduction. ALA has also been reported to regulate inflammation, oxidative stress, and cell proliferation in the testis and in cultured testicular cells [1,4]. For example, oral administration of ALA suppressed LPS-induced increases in NF-κB, COX-2, cPLA2, and iNOS in mouse testes and reduced serum IL-6 and TNF-α levels [5]. Under oxidative stress, 200 μM ALA increased superoxide dismutase (SOD) levels and the expression of *GPX4* and *Bcl2* in sheep Sertoli cells [6]. ALA also increased the expression of *Cyclin B1* and *Bcl2* in mouse TM4 cells and promoted cell proliferation [4]. In *Hu* lambs, feeding 8% flaxseed for 42 d increased testicular total antioxidant capacity (T-AOC) and SOD levels, promoted seminiferous tubule development, and increased cauda epididymal sperm count [7]. These studies suggest that ALA may have beneficial effects on testicular function and spermatogenesis.

Previous studies on the effects of ALA on the testes have mainly been conducted in vivo or using isolated testicular cells. In vivo, the effects of dietary ALA are influenced by digestion, rumen metabolism, absorption, and systemic regulation [8,9]. In fistulated sheep, 88% to 93% of dietary ALA was biohydrogenated in the rumen [10]. Stable isotope tracing in rats further showed that only 0.004% of orally administered ALA accumulated unchanged in the testis [11]. Therefore, the effects of ALA on testicular tissue remain difficult to evaluate in vivo. Isolated testicular cells can be used to examine the cellular effects of ALA, but they do not retain seminiferous tubule structure or interactions among testicular cells. Culture of testicular tissue fragments can preserve tissue structure and cellular function, providing an alternative approach to examining the direct response of intact testicular tissue to ALA [12,13,14]. However, the response of cultured sheep testicular tissue to ALA remains unclear.

This study aimed to evaluate the effects of ALA on seminiferous tubule structure and the spermatogonial population in cultured sheep testicular tissue fragments. Previous studies reported efficient utilization of ALA by testicular somatic cells at approximately 2.5–7.2 μM [2,15]. A lipid molecule at 5 μM has also been reported to promote germ cell proliferation in cultured sheep testicular tissue [14]. Therefore, sheep testicular tissue fragments were cultured on agarose for 50 d with 5 μM ALA. Histological analysis, UCHL1 immunofluorescence staining, and RNA sequencing were performed. This study may help clarify the effects of ALA on sheep testicular tissue and support further research on the nutritional regulation of male reproduction in ruminants.

## 2. Materials and Methods

### 2.1. Collection and Preparation of Testicular Tissue Fragments

All procedures involving animals were approved by the Animal Ethics and Welfare Committee of the Institute of Ruminant Research, Lanzhou University (approval no. CY20250712). Both testes were collected from one healthy 3-month-old *Hu* ram (*n* = 1) during routine castration at Xinyuan Sheep Farm in Honggu District, Lanzhou, China. The ram was group-housed in an indoor pen under commercial farm conditions, fed a standard commercial diet, and given free access to water. It had not received any dietary supplementation before castration. The ram tested negative for brucellosis before tissue collection. Castration was performed by an experienced veterinarian as part of routine farm management. Xylazine (0.15 mg/kg) was administered intramuscularly for sedation, and 2% lidocaine (4 mg/kg) was used for local anesthesia. The scrotum and surrounding skin were disinfected with povidone iodine before surgery and again after removal of the testes. Immediately after collection, the testes were placed in sterile Hanks’ balanced salt solution (Solarbio, Beijing, China) containing 1% penicillin–streptomycin (Gibco, Waltham, MA, USA). The samples were transported to the laboratory at 4 °C within 3 h. Under sterile conditions, the tunica albuginea, visible blood vessels, connective tissue, and damaged areas were removed. The testicular parenchyma was washed three times with sterile phosphate-buffered saline (PBS, Biosharp, Hefei, China) containing 1% penicillin–streptomycin. Tissue from both testes was combined and cut into fragments of approximately 2 mm^3^.

### 2.2. Preparation of Agarose Gel Supports

The agarose culture method was modified from a previously reported method for culturing testicular tissue fragments from livestock [12]. Agarose powder (Sigma-Aldrich, St. Louis, MO, USA, A9045) was dissolved in sterile distilled water to prepare a 1.5% (*w*/*v*) solution. The solution was autoclaved at 121 °C for 20 min and then poured into sterile culture dishes. After solidification, the agarose gel was cut into square blocks approximately 1 cm × 1 cm × 0.5 cm in size. One agarose block was placed in each well of a culture plate with six wells. The agarose blocks were immersed in complete culture medium and equilibrated for 24 h in a humidified incubator at 37 °C with 5% CO_2_.

### 2.3. Preparation of Culture Medium

Complete medium for sheep testicular tissue fragment culture was prepared using KnockOut™ DMEM (Gibco, Waltham, MA, USA, 10829018) as the basal medium. The complete medium formulation, including all supplements and their final concentrations, is provided in the Supplementary Materials. After preparation, the medium was filtered through a sterile 0.22 μm membrane filter and warmed to 37 °C before use.

### 2.4. Culture of Testicular Tissue Fragments and Sample Collection

**Figure 1:**
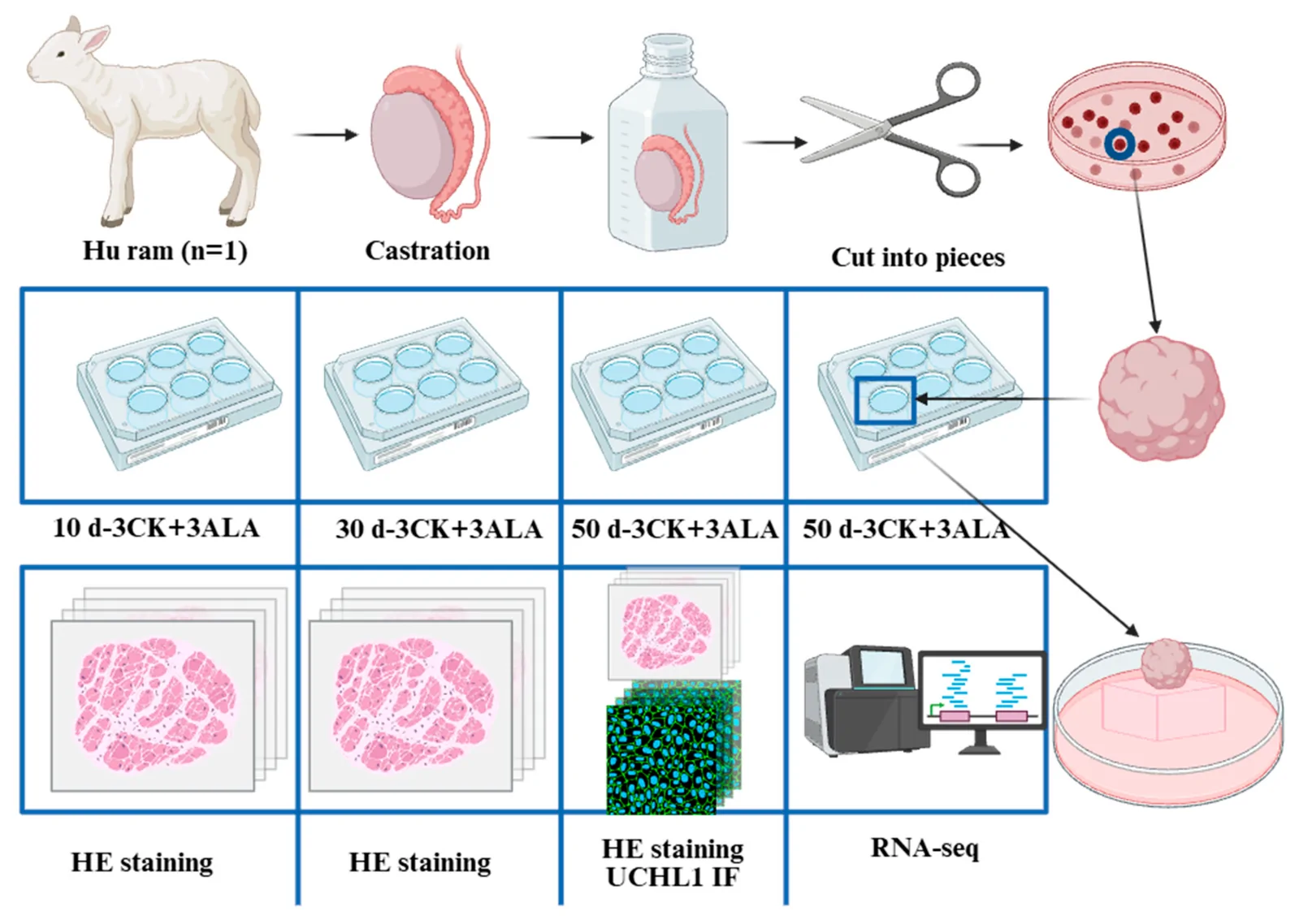
Experimental design of the study. Testicular tissue fragments were obtained from one 3-month-old *Hu* ram and manually allocated to four six-well plates. Each plate contained three CK and three ALA fragments. The four plates were cultured in parallel and used for histological analysis on days 10, 30, and 50 or RNA sequencing on day 50. UCHL1 immunofluorescence staining was performed on the day 50 histological samples. CK, control condition; ALA, α-linolenic acid condition.

All tissue fragments used in the experiment were obtained from the same ram. Before tissue placement, the equilibration medium was replaced with 1.5 mL of fresh complete medium. After tissue from both testes was combined and cut, the resulting fragments were thoroughly mixed. Fragments of similar size were then manually allocated to the CK and ALA conditions and distributed across the four six-well plates. One fragment was placed in each well, and each plate contained three CK and three ALA tissue fragments. Each tissue fragment cultured in an individual well was considered an experimental unit. Three plates were used for histological sampling on days 10, 30, and 50, respectively, and the fourth plate was used for RNA sequencing on day 50. The experimental design is shown in Figure 1. Based on previous studies [2,14,15] and our preliminary concentration screening, 5 μM ALA was selected for the subsequent experiment. ALA (MedChemExpress, Monmouth Junction, NJ, USA, HY-N0728) was dissolved in DMSO (MedChemExpress, Monmouth Junction, NJ, USA) to prepare a 10 mM stock solution. The stock solution was aliquoted and stored at −20 °C, protected from light. Each aliquot was thawed only once and used immediately. Following a previously described method [16], the ALA stock solution was diluted in complete medium to a final concentration of 5 μM. Fatty acid- and IgG-free BSA (MedChemExpress, Monmouth Junction, NJ, USA) was then added to a final concentration of 0.5%, and the mixture was incubated at 37 °C for 30 min to allow for ALA-BSA complex formation. The CK medium contained the same final concentrations of BSA and DMSO as the ALA medium. The final DMSO concentration was below 0.1% in both the CK and ALA media. The tissue fragments were cultured at 37 °C with 5% CO_2_ for up to 50 d. Culture of all four plates was initiated on the same day, and the plates were maintained in the same incubator under identical culture conditions. The medium was replaced every 3 d with freshly prepared CK or ALA medium. A fresh tissue fragment collected before culture was fixed and used as the day 0 sample. On days 10, 30, and 50, all tissue fragments from the corresponding plates were collected and fixed in Animal Testicular Tissue Fixative Solution (Servicebio, Wuhan, China, G1121) for histological analysis. The day 50 histological samples were also used for UCHL1 immunofluorescence staining. All tissue fragments from the plate used for RNA sequencing were collected on day 50, immediately frozen in liquid nitrogen, and stored at −80 °C until analysis.

### 2.5. Histological Analysis

Following fixation for 24 h at room temperature, the tissue fragments were dehydrated through a graded ethanol series consisting of 70%, 80%, 90%, 95%, and 100% ethanol. The tissues were then cleared in xylene, infiltrated with molten paraffin, and embedded in paraffin. Serial sections were cut at a thickness of 5 μm using a rotary microtome (Leica Instruments, Shanghai, China, RM2016) and mounted on glass slides. The sections were deparaffinized in xylene and rehydrated through a descending ethanol series. The sections were then stained with hematoxylin and eosin (HE). After staining, the sections were dehydrated through a graded ethanol series, cleared in xylene, and mounted with neutral balsam.

Images were captured using an Olympus IX71 microscope v3.11 (Olympus, Tokyo, Japan). Three independently cultured tissue fragments per condition were analyzed at each time point. One section was analyzed from each fragment, and five fields were selected from each section. Only seminiferous tubules with an approximately circular profile, defined as a major-to-minor axis ratio < 2, were included in the analysis [17]. Approximately 15–30 seminiferous tubules were measured per fragment. For each tubule, the major axis and the perpendicular minor axis passing through the center were measured, and their mean was used as the seminiferous tubule diameter. Measurements from all selected tubules were averaged to obtain one value for each tissue fragment.

The same seminiferous tubules used for diameter measurements were analyzed for empty space. Empty space was quantified using Fiji/ImageJ v1.54p (National Institutes of Health, Bethesda, MD, USA) with a custom Jython script. Within each tubule area, pixels with saturation ≤ 31/255 and brightness ≥ 242/255 were classified as empty space [18,19]. Adjacent empty space pixels were grouped using eight-connectivity, and connected regions smaller than 5 μm^2^ were excluded. The segmentation results were inspected against the original HE images for quality control. The empty space area fraction was calculated as the total area of accepted empty space regions divided by the corresponding seminiferous tubule area and multiplied by 100%. Image selection, seminiferous tubule delineation, and morphometric measurements were performed by an investigator who was blinded to treatment allocation.

### 2.6. Immunofluorescence Staining

Paraffin sections were deparaffinized and rehydrated. Antigen retrieval was performed in 0.01 M sodium citrate buffer (Servicebio, Wuhan, China; pH 6.0) at 96 °C for 15 min, followed by cooling to room temperature. The sections were blocked with 5% BSA for 1 h at room temperature and incubated overnight at 4 °C with a rabbit anti-UCHL1 primary antibody (1:200, Servicebio, Wuhan, China, GB150176). The specificity of UCHL1 immunostaining in *Hu* sheep testicular tissue has been confirmed by peptide blocking assays [20,21]. After washing three times with PBS for 5 min each, the sections were incubated with FITC-conjugated donkey anti-rabbit IgG (H + L) secondary antibody (1:200, Servicebio, Wuhan, China, GB22403) for 1 h at 37 °C in the dark. After washing three times with PBS, nuclei were stained with DAPI (Servicebio, Wuhan, China) for 10 min. For the negative control, the primary antibody was omitted. Images were captured using an Olympus IX71 microscope under the same acquisition settings. Three independently cultured tissue fragments per condition were analyzed. One section was examined from each tissue fragment, and three random fields were selected from each section. Five seminiferous tubules were evaluated in each field, for a total of 15 tubules per fragment. UCHL1-positive cells were counted within each seminiferous tubule using ImageJ software. The mean number of UCHL1-positive cells per seminiferous tubule was calculated for each tissue fragment.

### 2.7. RNA Extraction, Library Construction, and Sequencing

Total RNA was extracted separately from six tissue fragments collected on day 50, including three CK fragments and three ALA-treated fragments. Each fragment was cultured in a separate well, and all six fragments were obtained from the same ram. RNA was extracted using TRIzol reagent (TIANGEN Biotech, Beijing, China) according to the manufacturer’s instructions. RNA concentration and purity were measured using a NanoDrop 2000 spectrophotometer (Thermo Fisher Scientific, Waltham, MA, USA). RNA integrity was assessed using an Agilent 2100 Bioanalyzer (Agilent Technologies, Santa Clara, CA, USA). Samples with at least 1 μg of total RNA, an RNA concentration of at least 100 ng/μL, an A260/A280 ratio of 1.8 to 2.0, and an RNA integrity number of at least 7 were used for library preparation. mRNA libraries were prepared using the VAHTS Universal V6 RNA-seq Library Prep Kit for Illumina (Vazyme Biotech Co., Ltd., Nanjing, China) according to the manufacturer’s instructions. Poly(A)+ RNA was enriched using oligo(dT) magnetic beads and fragmented into short fragments. First-strand cDNA was synthesized from the fragmented RNA using random hexamer primers, followed by second-strand cDNA synthesis. The subsequent steps included end repair and dA-tailing, sequencing adapter ligation, fragment size selection, and PCR amplification. Library quality was assessed using an Agilent 2100 Bioanalyzer (Agilent Technologies, Santa Clara, CA, USA). Qualified libraries were sequenced by OE Biotech Co., Ltd. (Shanghai, China) on an Illumina NovaSeq 6000 platform (Illumina, San Diego, CA, USA) to generate 150 bp paired-end reads.

### 2.8. RNA Sequencing Data Processing and Differential Expression Analysis

Raw sequencing reads were processed using fastp v0.20.1 to remove adapter sequences and low-quality reads. Q30 values and GC content were calculated from the clean reads. Clean reads were aligned to the sheep reference genome ARS-UI_Ramb_v2.0 (GCF_016772045.1) using HISAT2 v2.1.0 with reverse-strand specificity. Gene-level read counts were generated using HTSeq-count v0.11.2 with the reverse-stranded setting, based on the corresponding NCBI genome annotation (GCF_016772045.1_ARS-UI_Ramb_v2.0_genomic.gff). Genes with raw counts ≥ 10 in at least three of the six libraries were retained for subsequent analysis, resulting in 15,809 genes. Sequencing quality was evaluated based on the number of clean reads, Q30 values, mapping rates, and gene-level read counts. Differential expression analysis between the CK and ALA-treated fragments was performed using DESeq2 v1.52.0 in R v4.6.1. DESeq2 uses a negative binomial generalized linear model to test differences in gene expression between the two culture conditions. *p* values were adjusted using the Benjamini–Hochberg method to control the false discovery rate (FDR). Genes with FDR < 0.05 and |log_2_FC| ≥ 1 were defined as differentially expressed genes (DEGs). Expression values in the heatmaps were standardized as row z scores.

### 2.9. Gene Set Enrichment Analysis

Gene set enrichment analysis (GSEA) was conducted using ranked gene lists derived from the DESeq2 results. GO-GSEA and KEGG-GSEA were performed separately for the ALA versus CK comparison using fgseaMultilevel in fgsea v1.38.0 in R v4.6.1. Genes were ranked by the Wald statistic obtained from DESeq2. Gene sets containing 15 to 500 genes were included, and fgseaMultilevel was run with eps = 0. Gene sets with an FDR < 0.05 were considered significantly enriched. Representative gene sets were selected for visualization based on statistical significance and biological relevance. The top 10 leading-edge genes from each selected gene set were used for heatmap visualization. Expression values in the heatmaps were standardized as row z scores.

### 2.10. Cell-Type-Associated Transcriptional Signature Analysis

Cell-type marker genes were obtained from a published single-cell RNA sequencing study of 3-month-old *Hu* sheep testes [22]. Marker genes for 14 annotated testicular cell types were included, with up to the top 100 markers selected for each cell type. Duplicate genes, ribosomal protein genes, mitochondrial genes, hemoglobin genes, and *MALAT1* were removed. Marker names were matched without regard for case to gene symbols in the RNA-seq data generated in this study. When no gene symbol match was found, an exact match to the annotation ID was used as a fallback. Unmatched markers were excluded. The matched markers were then used to define the gene set for each cell type. Genes were ranked by the Wald statistic obtained from DESeq2, and preranked GSEA was performed using fgseaMultilevel in fgsea v1.38.0. Gene sets containing 15 to 200 matched marker genes were included in the analysis. Two sensitivity analyses were also performed. The first used the top 50 marker genes for each cell type, and the second used marker genes unique to a single annotated cell type. Multiple testing correction was performed separately for each marker selection strategy.

### 2.11. Quantitative Real-Time PCR Analysis

The same RNA samples used for RNA-seq library construction were also used for qRT-PCR. A total of 0.5 μg RNA was reverse transcribed into cDNA using EasyScript^®^ One-Step gDNA Removal and cDNA Synthesis SuperMix (TransGen Biotech, Beijing, China) at 42 °C for 15 min. The cDNA was diluted 1:20 with nuclease-free water before qPCR. Gene expression was measured using a CFX384 Touch™ Real-Time PCR Detection System (Bio-Rad, Hercules, CA, USA) with PerfectStart^®^ Green qPCR SuperMix (TransGen Biotech, Beijing, China). Each 20 μL reaction contained 10 μL of 2× PerfectStart Green qPCR SuperMix, 5 μL of diluted cDNA, 0.5 μL each of forward and reverse primers, and 4 μL of nuclease-free water. The amplification conditions were 95 °C for 5 min, followed by 40 cycles of 95 °C for 5 s and 61 °C for 30 s. Melting curve analysis was performed to confirm amplification specificity. Relative gene expression was normalized to *HPRT1* and calculated using the 2^−ΔΔCt^ method. Primer sequences are listed in Table 1.

### 2.12. Statistical Analysis

Data are presented as mean ± SEM. Histological and UCHL1 immunofluorescence data were summarized using descriptive statistics only. qRT-PCR data were analyzed using two-sided Welch’s *t*-tests for exploratory comparisons at the fragment level. Statistical analyses were performed using SPSS v18.0 (IBM Corporation, Armonk, NY, USA), and graphs were generated using GraphPad Prism v8.0 (GraphPad Software, San Diego, CA, USA). * *p* < 0.05 and ** *p* < 0.01 were considered statistically significant.

## 3. Results

### 3.1. Seminiferous Tubule Morphology in Cultured Sheep Testicular Tissue Fragments

**Figure 2:**
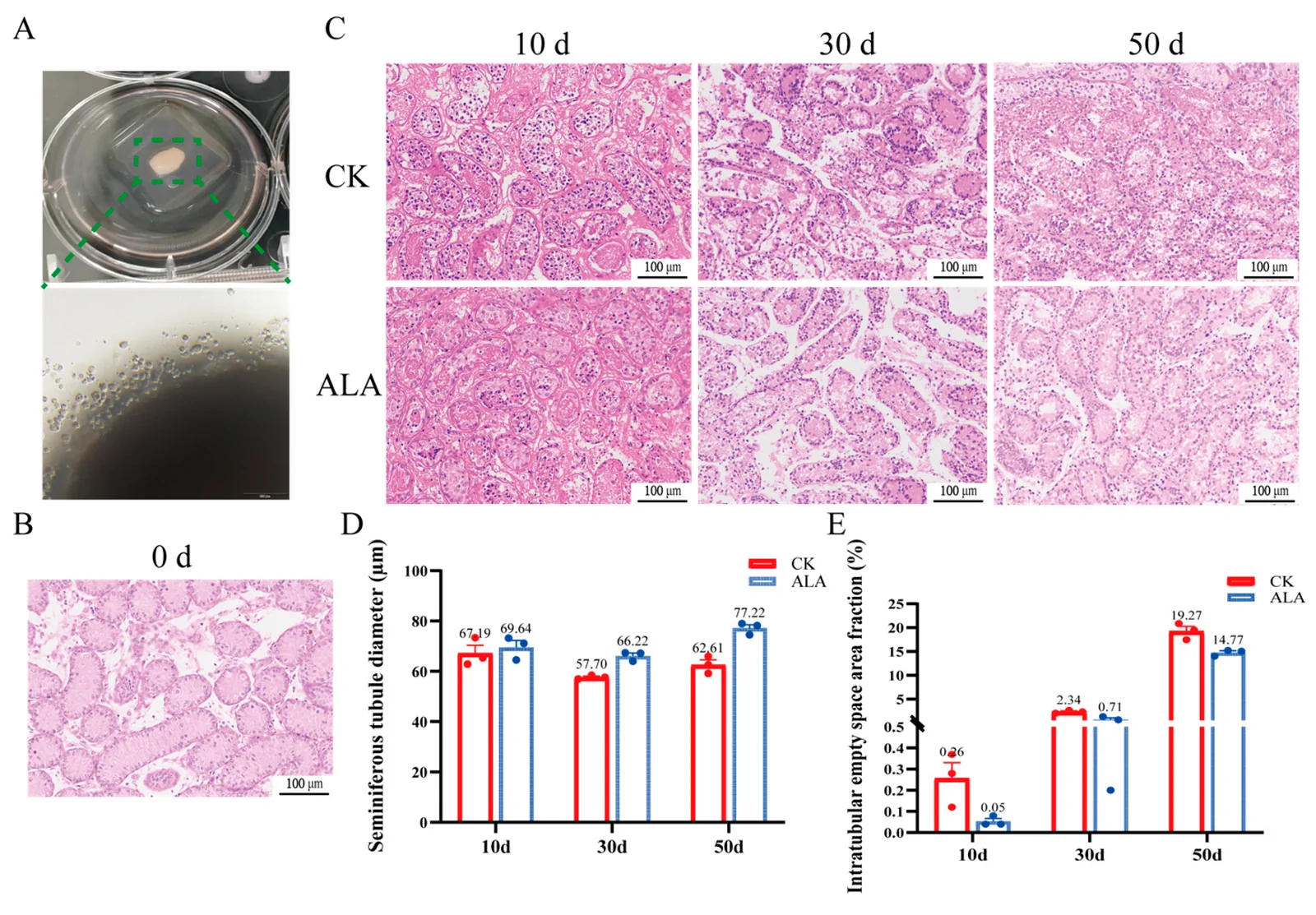
Morphological changes in cultured sheep testicular tissue fragments after ALA treatment. (**A**) Representative photograph and bright-field image of sheep testicular tissue fragments cultured on agarose. (**B**) Representative section of sheep testicular tissue before culture. The day-0 sample is shown as a representative baseline and was not included in the quantitative analysis. (**C**) Representative sections from CK and ALA-treated fragments on days 10, 30, and 50. (**D**) Seminiferous tubule diameter on days 10, 30, and 50. (**E**) Proportion of empty space within the seminiferous tubules on days 10, 30, and 50. Images in panels (**B**,**C**) were captured using a 20× objective lens, corresponding to 200× total magnification. Scale bar = 100 μm. Data are presented as mean ± SEM. Points represent independently cultured tissue fragments. Three fragments per condition were analyzed at each time point. One section and five fields were analyzed per fragment, with approximately 15–30 seminiferous tubules measured per fragment. All fragments were obtained from the same ram. CK, control. ALA, α-linolenic acid treatment.

Sheep testicular tissue fragments were cultured on agarose (Figure 2A). Before culture, the tissue fragments contained round or oval seminiferous tubules with densely arranged cells (Figure 2B). The mean seminiferous tubule diameter was similar between the CK and ALA fragments on day 10 (67.19 vs. 69.64 μm) and was greater in the ALA-treated fragments on days 30 (57.70 vs. 66.22 μm) and 50 (62.61 vs. 77.22 μm, Figure 2C,D). The mean proportion of empty space within the seminiferous tubules was low in both conditions on day 10 (0.26% vs. 0.05%) and was lower in ALA-treated fragments on days 30 (2.34% vs. 0.71%) and 50 (19.27% vs. 14.77%; Figure 2E).

### 3.2. Number of UCHL1-Positive Cells per Seminiferous Tubule

**Figure 3:**
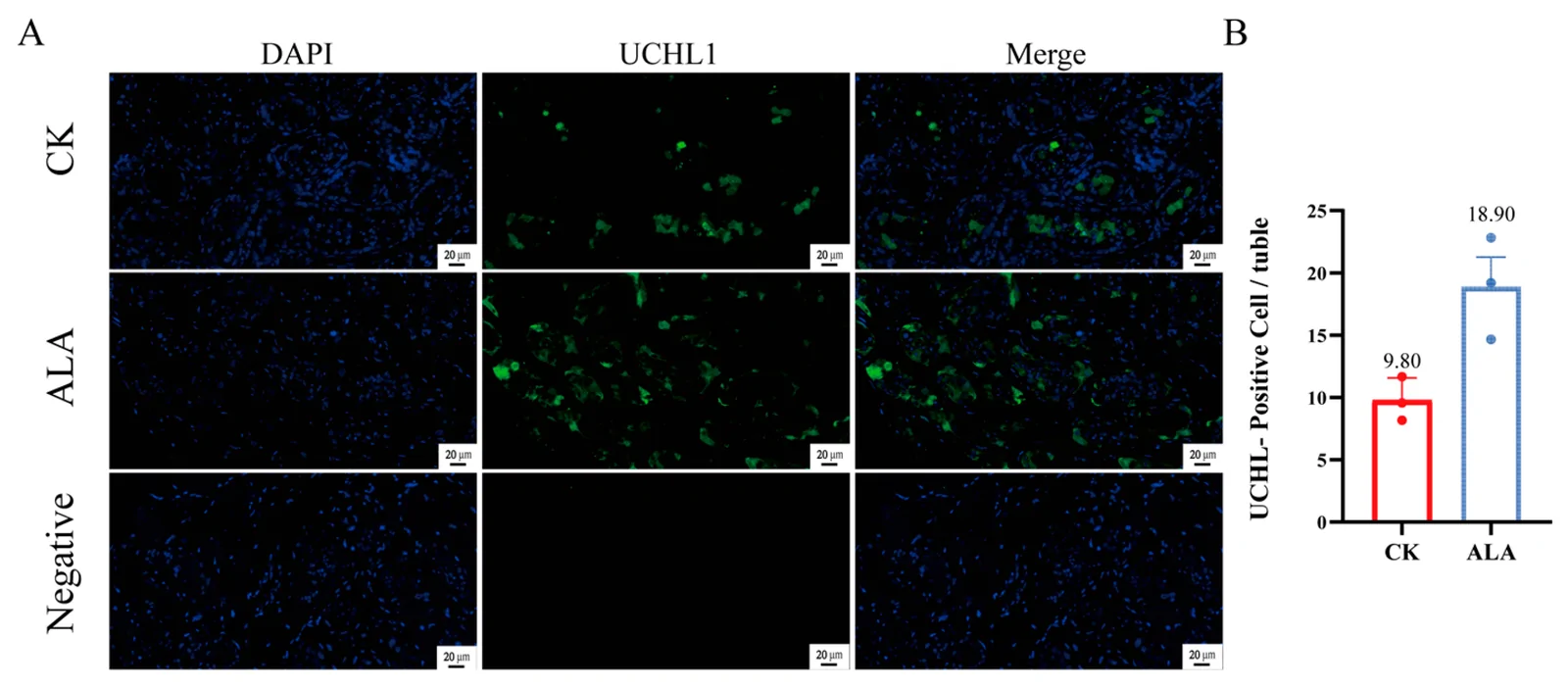
UCHL1-positive cells per seminiferous tubule in cultured sheep testicular tissue fragments after ALA treatment. (**A**) Representative immunofluorescence images of UCHL1 staining in CK and ALA-treated fragments and the negative control (primary antibody omitted). UCHL1 staining is shown in green, and nuclei stained with DAPI are shown in blue. Images were captured using a 40× objective lens. Scale bar = 20 μm. (**B**) Number of UCHL1-positive cells per seminiferous tubule. Data are presented as mean ± SEM. Points represent independently cultured tissue fragments. Three fragments per condition were analyzed. One section and three fields were examined per fragment, with five seminiferous tubules evaluated per field and 15 seminiferous tubules evaluated per fragment. All fragments were obtained from the same ram. CK, control. ALA, α-linolenic acid treatment.

UCHL1-positive cells were present within the seminiferous tubules in CK and ALA-treated fragments (Figure 3A). No UCHL1 fluorescence signal was observed in the negative control. The mean number of UCHL1-positive cells per seminiferous tubule was higher in the ALA-treated fragments than in the CK fragments (18.90 vs. 9.80 cells/tubule, Figure 3B).

### 3.3. RNA Sequencing and Differential Gene Expression Analysis

**Figure 4:**
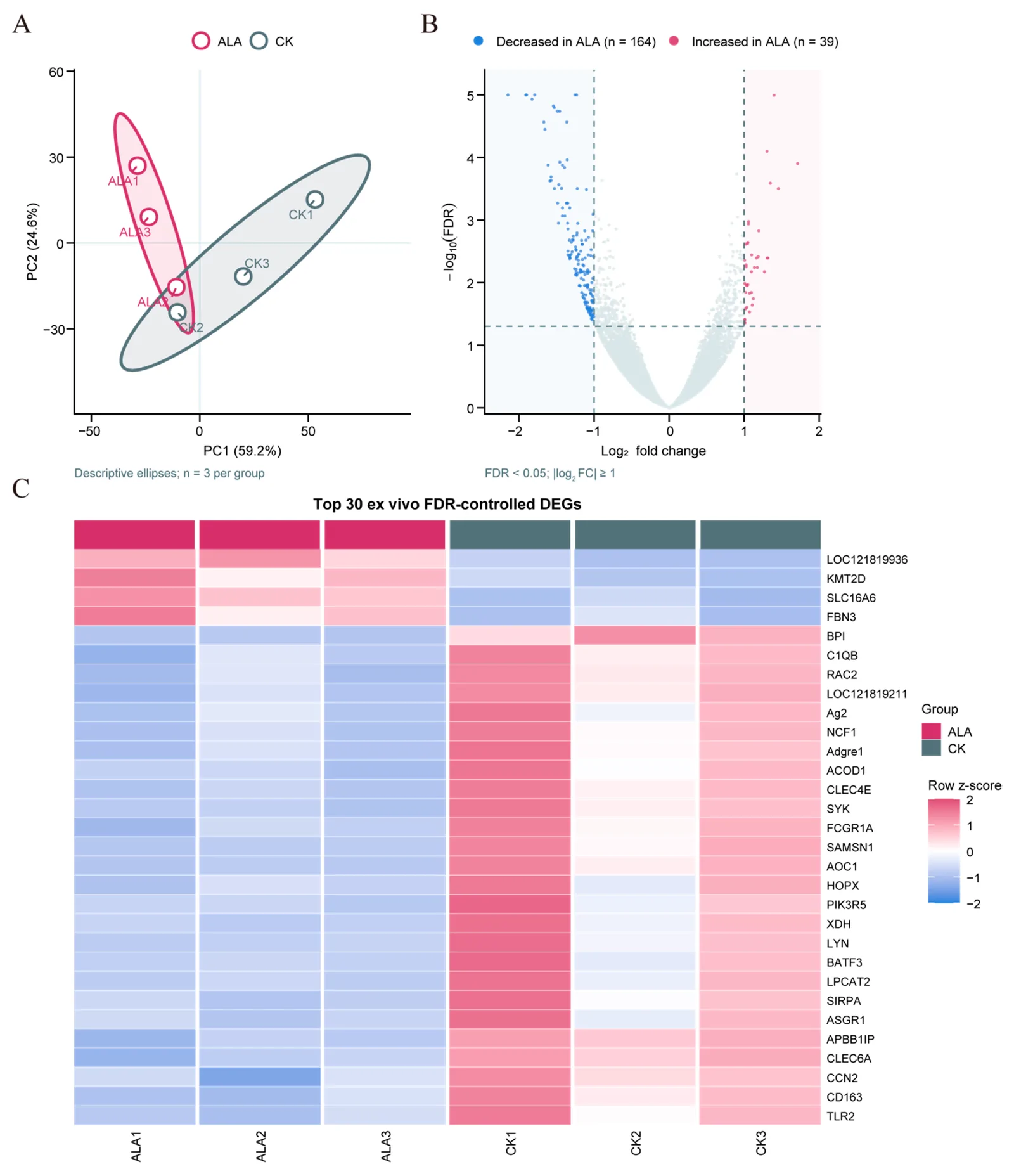
Gene expression changes in cultured sheep testicular tissue fragments after ALA treatment. (**A**) PCA score plot of the CK and ALA-treated fragments. PC1 and PC2 explained 59.2% and 24.6% of the variation, respectively. (**B**) Volcano plot of DEGs between the CK and ALA-treated fragments. Red and blue dots indicate genes upregulated and downregulated in the ALA-treated fragments, respectively, and gray dots indicate genes that did not meet the thresholds. The light red background shows log_2_FC ≥ 1, and the light blue background shows log_2_FC ≤ −1. The vertical dashed lines mark log_2_FC = −1 and 1. The horizontal dashed line marks FDR = 0.05. (**C**) Heatmap of the top 30 DEGs. The 203 DEGs were ranked by FDR from low to high. Genes with the same FDR were ranked by |log_2_FC| from high to low, and the first 30 genes were selected for the heatmap. Expression values are shown as row z scores. Three independently cultured tissue fragments per condition were analyzed. Each fragment was cultured in a separate well and used as one RNA-seq sample. All fragments were obtained from the same ram. CK, control. ALA, α-linolenic acid treatment.

RNA sequencing was performed on three independently cultured tissue fragments per condition. All six fragments were obtained from the same ram. RNA sequencing generated 41.84 to 52.40 million clean reads per library. Q30 values ranged from 91.60% to 92.07%, and the mapping rates to the sheep reference genome ranged from 97.55% to 98.05% (Table S1). Principal component analysis (PCA) showed separation between the CK and ALA-treated fragments mainly along the first principal component (PC1), which explained 59.2% of the total variance. The second principal component (PC2) explained 24.6% of the variance (Figure 4A). A total of 203 DEGs were identified at FDR < 0.05 and |log_2_FC| ≥ 1. Among them, 39 genes were upregulated and 164 were downregulated in the ALA-treated fragments relative to the CK fragments (Figure 4B). The top 30 DEGs showed different expression patterns between the CK and ALA-treated fragments (Figure 4C, Table S2).

### 3.4. GO and KEGG Gene Set Enrichment Analysis

**Figure 5:**
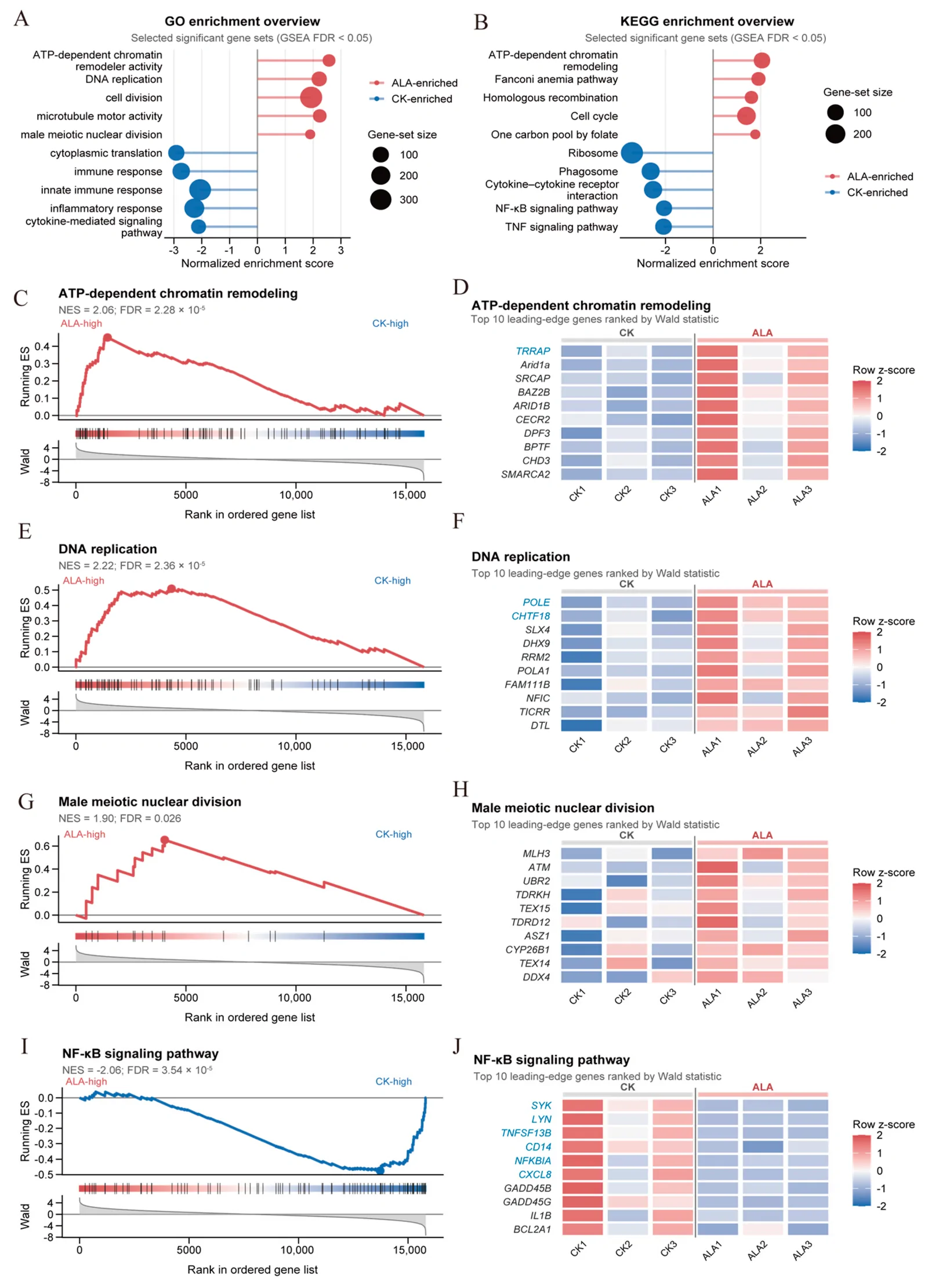
GO and KEGG gene set enrichment analysis (GSEA) of cultured sheep testicular tissue fragments after ALA treatment. (**A**) Selected significant GO terms identified by GSEA. (**B**) Selected significant KEGG pathways identified by GSEA. Positive normalized enrichment score (NES) values indicate enrichment in the ALA-treated fragments, whereas negative NES values indicate enrichment in the CK fragments. Dot size indicates gene set size. (**C**) GSEA plot of ATP-dependent chromatin remodeling. (**D**) Heatmap of the top 10 leading-edge genes for ATP-dependent chromatin remodeling. (**E**) GSEA plot of DNA replication. (**F**) Heatmap of the top 10 leading-edge genes for DNA replication. (**G**) GSEA plot of male meiotic nuclear division. (**H**) Heatmap of the top 10 leading-edge genes for male meiotic nuclear division. (**I**) GSEA plot of the NF-κB signaling pathway. (**J**) Heatmap of the top 10 leading-edge genes for the NF-κB signaling pathway. In panels (**C**,**E**,**G**,**I**), the red and blue lines show the running enrichment score for ALA and CK, respectively. Black vertical lines indicate the positions of genes in the gene set. The red-to-blue bar shows the ranked gene list from ALA-high to CK-high, and the gray area shows the ranked list metric. The leading-edge genes in each gene set were ranked by the DESeq2 Wald statistic according to the direction of enrichment, and the top 10 genes were selected. Gene names shown in blue indicate DEGs with an FDR < 0.05 and |log_2_FC| ≥ 1. Gene sets with an FDR < 0.05 were considered significant. Three independently cultured tissue fragments per condition were analyzed. All fragments were obtained from the same ram. CK, control. ALA, α-linolenic acid treatment.

To identify coordinated pathway changes across all ranked genes, GSEA was performed using GO and KEGG gene sets. GO-GSEA showed that ATP-dependent chromatin remodeler activity, DNA replication, cell division, microtubule motor activity, and male meiotic nuclear division were enriched in the ALA-treated fragments. Cytoplasmic translation, immune response, innate immune response, inflammatory response, and the cytokine-mediated signaling pathway were enriched in the CK fragments (Figure 5A, Table S3). KEGG-GSEA showed that ATP-dependent chromatin remodeling, the Fanconi anemia pathway, homologous recombination, cell cycle, and one carbon pool by folate were enriched in the ALA-treated fragments. The ribosome, the phagosome, cytokine–cytokine receptor interaction, the NF-κB signaling pathway, and the TNF signaling pathway were enriched in the CK fragments (Figure 5B, Table S3). Among these gene sets, ATP-dependent chromatin remodeling (NES = 2.06, FDR < 0.01), DNA replication (NES = 2.22, FDR < 0.01), and male meiotic nuclear division (NES = 1.90, FDR < 0.05) were enriched in the ALA-treated fragments, whereas the NF-κB signaling pathway was enriched in the CK fragments (NES = −2.06, FDR < 0.01, Figure 5C,E,G,I). The expression patterns of the top 10 leading-edge genes for these gene sets are shown in the heatmaps. Among these genes, the DEGs *TRRAP*, *POLE*, and *CHTF18*, which are involved in ATP-dependent chromatin remodeling and DNA replication, showed higher expression in the ALA-treated fragments. In contrast, *SYK*, *LYN*, *TNFSF13B*, *CD14*, *NFKBIA* and *CXCL8* in the NF-κB signaling pathway showed higher expression in the CK fragments.

### 3.5. Cell-Type Marker Gene Set Enrichment Analysis

**Figure 6:**
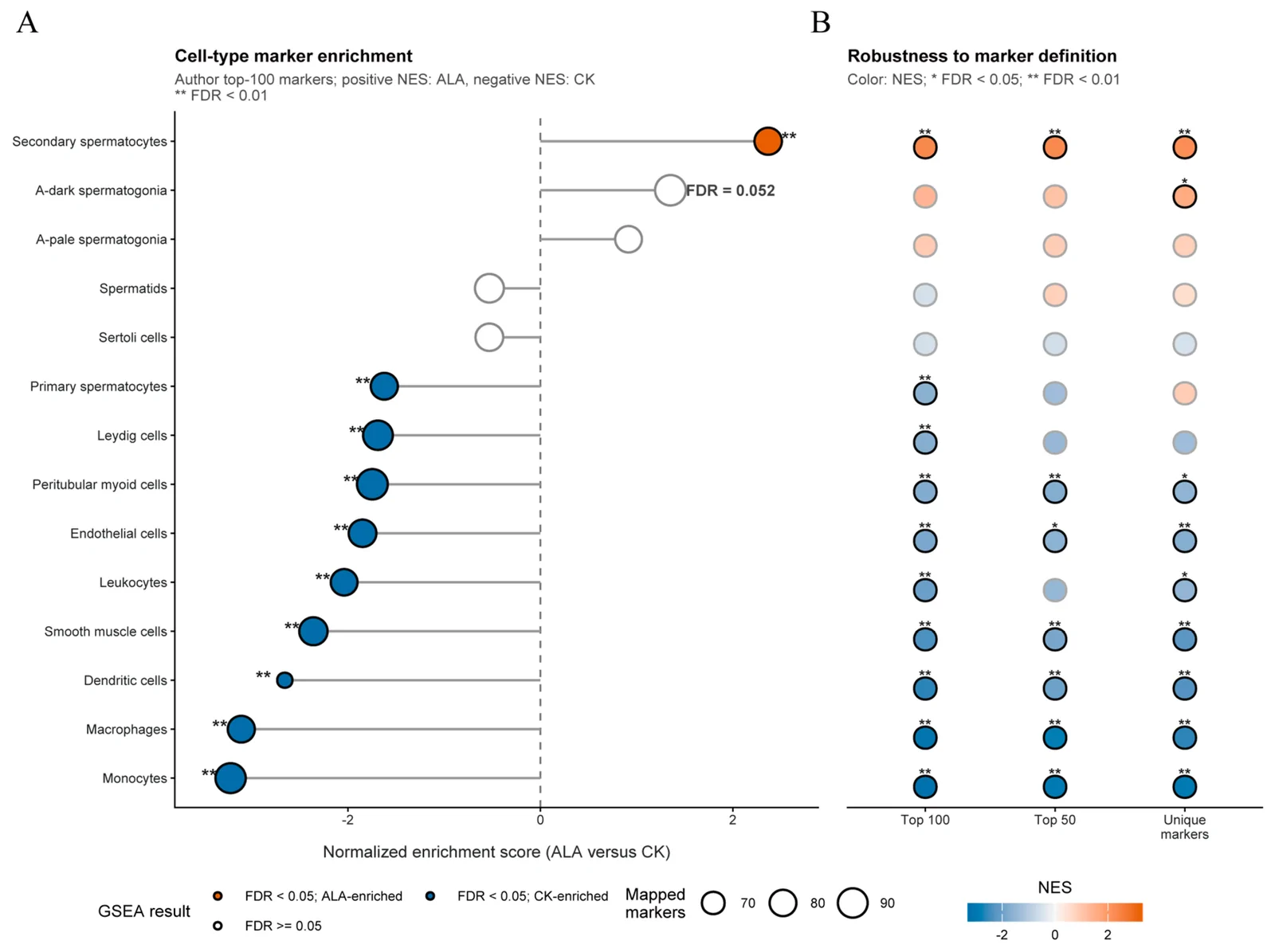
Cell-type marker gene set enrichment in cultured sheep testicular tissue fragments after ALA treatment. (**A**) GSEA results based on the top 100 marker genes for 14 annotated testicular cell types. Positive normalized enrichment score (NES) values indicate relative enrichment of the corresponding marker gene set in the ALA-treated fragments, whereas negative NES values indicate relative enrichment in the CK fragments. Dot size indicates the number of marker genes matched to the RNA-seq data generated in this study. Filled dots indicate FDR < 0.05. The exact FDR is shown for the A-dark spermatogonia marker gene set. (**B**) Sensitivity analyses based on the top 100 markers, top 50 markers, and markers unique to a single annotated cell type. Dot color indicates the NES. FDR values were adjusted separately across the 14 marker gene sets for each marker definition. * FDR < 0.05, ** FDR < 0.01. Three independently cultured tissue fragments per condition were analyzed. All fragments were obtained from the same ram. CK, control. ALA, α-linolenic acid treatment.

The secondary spermatocyte marker gene set was enriched in the ALA-treated fragments, whereas macrophage, monocyte, and other cell-type marker gene sets were enriched in the CK fragments (FDR < 0.05, Figure 6A). The A-dark spermatogonia marker gene set showed a trend toward enrichment in the ALA-treated fragments (FDR = 0.052). Similar results were obtained using the top 50 markers and markers unique to a single annotated cell type, supporting the robustness of the results (Figure 6B, Table S4).

### 3.6. Verification of DEGs by qRT-PCR

**Figure 7:**
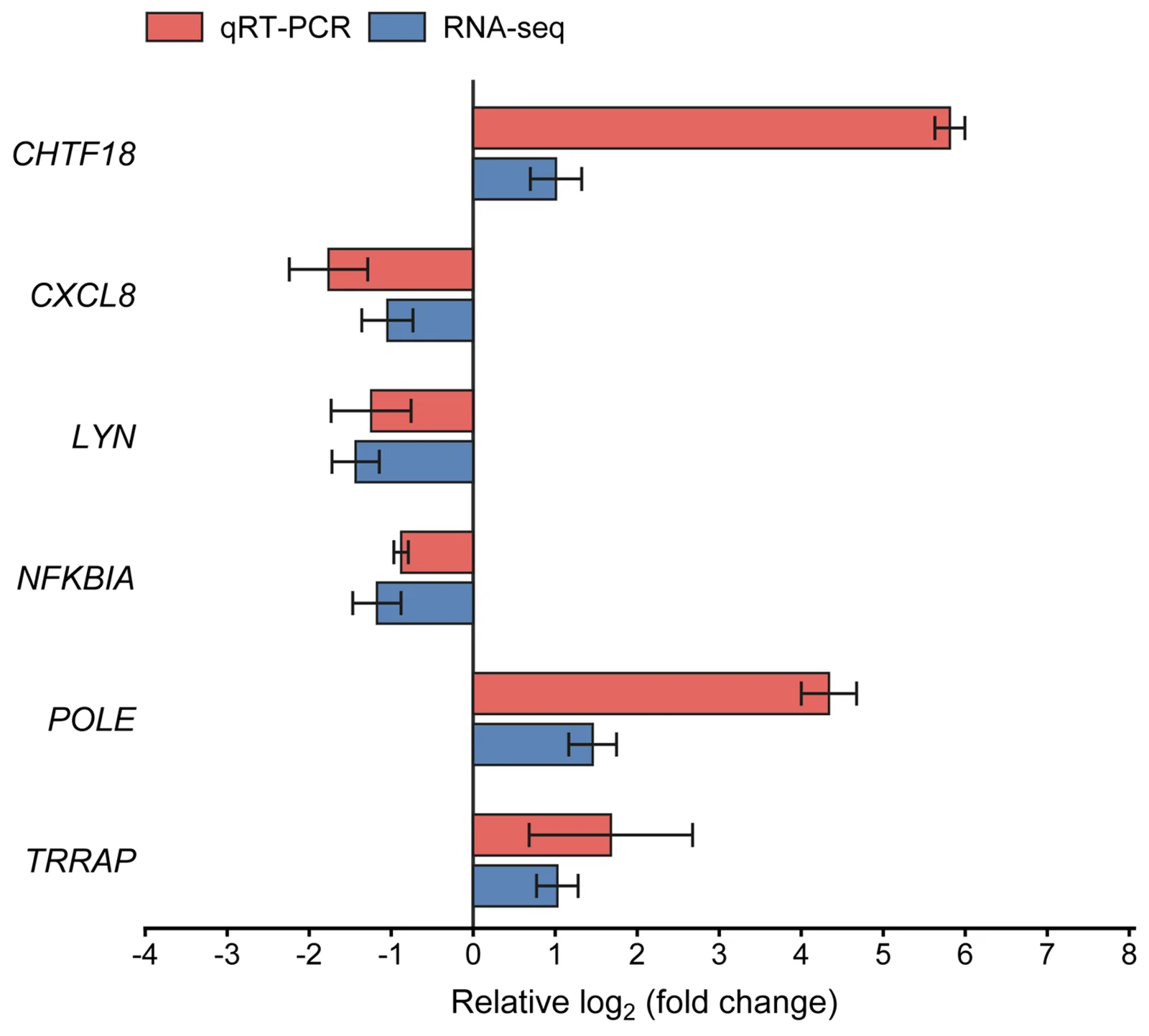
Validation of differentially expressed genes by qRT-PCR. The expression levels of *CHTF18*, *CXCL8*, *LYN*, *NFKBIA*, *POLE*, and *TRRAP* were measured by qRT-PCR and compared with the RNA-seq results. Red bars represent qRT-PCR results, and blue bars represent RNA-seq results. All six genes showed the same direction of change between the two methods. Data are presented as mean ± SEM. Three fragments per condition were analyzed. All fragments were obtained from the same ram. CK, control. ALA, α-linolenic acid treatment.

The expression levels of six DEGs (*CHTF18*, *CXCL8*, *LYN*, *NFKBIA*, *POLE*, and *TRRAP*) were analyzed using qRT-PCR. The expression of *CHTF18*, *POLE*, and *TRRAP* was higher in the ALA-treated fragments, whereas *CXCL8*, *LYN*, and *NFKBIA* showed lower expression. These changes were consistent with the RNA-seq data (Figure 7).

### 3.7. Expression of Germ Cell and Proliferation Markers

**Figure 8:**
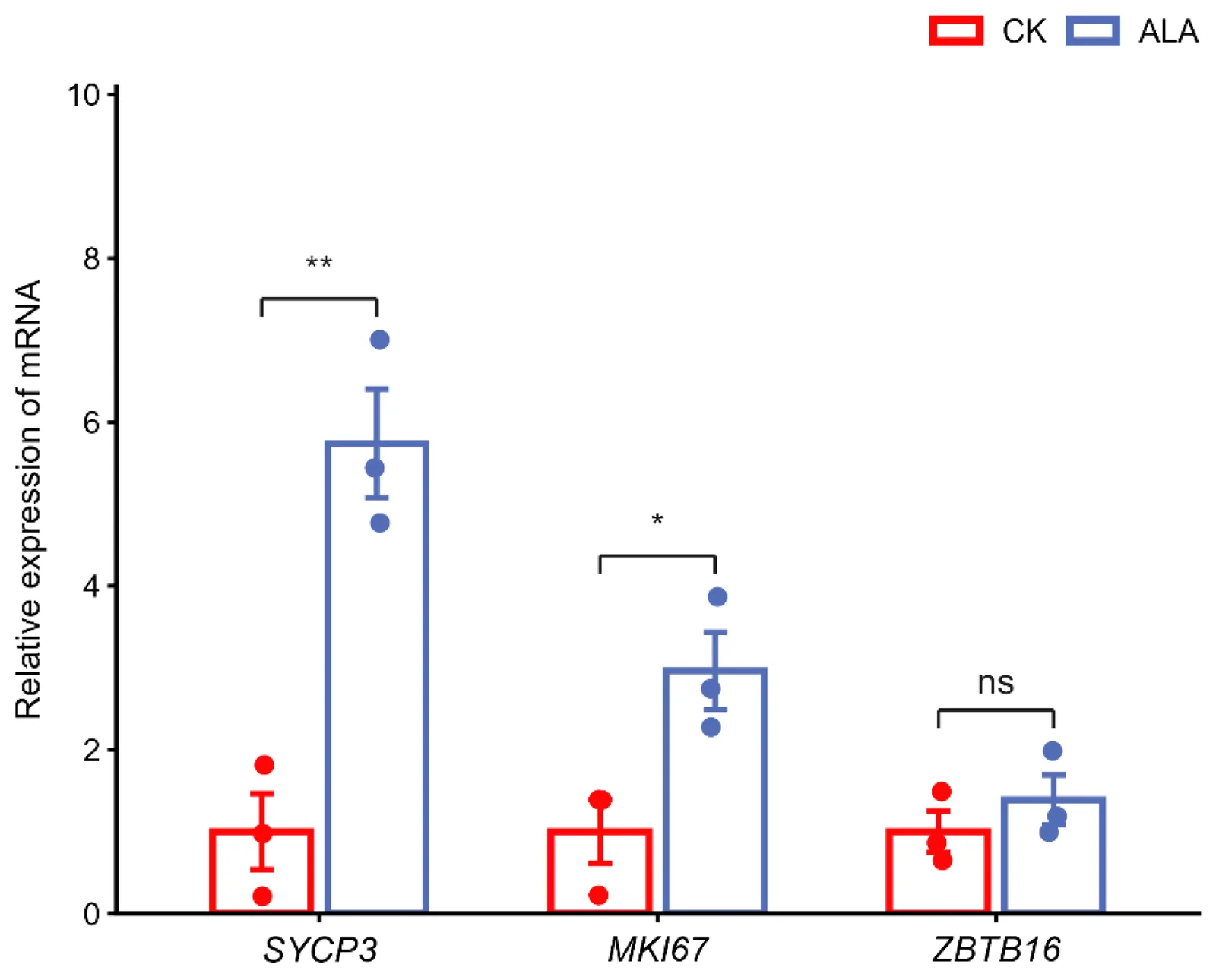
Relative expression levels of *SYCP3*, *MKI67*, and *ZBTB16* in cultured sheep testicular tissue fragments. The expression levels of *SYCP3*, *MKI67*, and *ZBTB16* were measured by qRT-PCR. Data are presented as mean ± SEM. Points represent independently cultured tissue fragments. Three fragments per condition were analyzed. All fragments were obtained from the same ram. * *p* < 0.05; ** *p* < 0.01. ns, not significant. CK, control. ALA, α-linolenic acid treatment.

The relative expression levels of *SYCP3* and *MKI67* in the ALA-treated fragments were significantly higher than those in the CK fragments (*p* < 0.05, Figure 8), whereas the relative expression level of *ZBTB16* did not differ between the two culture conditions (*p* > 0.05).

## 4. Discussion

In this study, ALA-treated fragments showed a greater seminiferous tubule diameter on culture days 30 and 50. During normal testicular development, seminiferous tubule diameter generally increases with germ cell proliferation and differentiation [20]. Larger seminiferous tubules during in vitro culture have also been associated with higher numbers of undifferentiated spermatogonia and meiotic germ cells [23,24]. However, an increase in seminiferous tubule diameter does not always reflect improved germ cell development, because germ cell degeneration may still occur during culture [25]. In addition, luminal fluid accumulation and increased pressure can cause seminiferous tubule dilation and may be accompanied by germ cell vacuolation or tubular atrophy [26,27]. Therefore, the greater seminiferous tubule diameter in fragments treated with ALA may be related to tubule growth, but changes in the tubular lumen during culture cannot be excluded. In contrast, increased empty space within seminiferous tubules is generally associated with tissue damage [27]. It may reflect fluid accumulation, enlarged intercellular spaces, Sertoli cell injury, or germ cell loss [27,28]. In cultured human testicular tissue, increased empty space within the seminiferous tubules was accompanied by a decrease in spermatocytes, germ cell loss, and disorganization of the seminiferous epithelium [29]. Therefore, the lower proportion of empty space within the seminiferous tubules in the ALA-treated fragments may be associated with better preservation of intratubular cells. Consistent with this finding, ALA-treated fragments contained more UCHL1-positive cells per seminiferous tubule. UCHL1 is commonly used as a marker of undifferentiated spermatogonia [30]. In *Hu* sheep, the number of UCHL1-positive cells increased after 30 days of age and reached a high level at 180 days, and these cells were also PCNA-positive from 90 days of age, indicating proliferative activity [20]. UCHL1 is also involved in spermatogonial maintenance, and UCHL1 knockdown has been shown to inhibit the proliferation of spermatogonial stem cells and increase apoptosis [31]. Therefore, the greater number of UCHL1-positive cells in the ALA-treated fragments may be associated with a greater abundance of undifferentiated spermatogonia. A previous study also showed that a diet rich in ALA from linseed increased testicular PCNA expression and epididymal sperm count in *Hu* lambs [7]. However, all tissue fragments in the present study were obtained from the same ram. Therefore, these morphological and cellular differences need to be confirmed using tissues from additional rams.

In addition to these morphological and cellular differences, the exploratory transcriptomic analysis showed different enrichment of inflammation related gene sets between the CK and ALA-treated fragments. Cell loss and basement membrane rupture have been reported in cultured testicular tissue fragments [13]. Because cultured tissue fragments lack blood perfusion, limited supplies of oxygen and nutrients may induce cellular stress and tissue damage, thereby promoting the inflammatory responses commonly observed during long-term testicular tissue culture [32]. TLR4-NF-κB signaling and macrophage activation have been shown to contribute to inflammatory responses during testicular tissue fragment culture [33]. Sustained NF-κB activation in the testis increases the production of proinflammatory factors, disrupts seminiferous tubule structure, and impairs spermatogenesis [34]. Inhibition of this pathway can reduce germ cell apoptosis [35]. Consistent with these findings, gene sets related to immune response, inflammatory response, and cytokine-mediated signaling were enriched in the CK fragments. The NF-κB signaling pathway was also enriched in the CK fragments. The expression of *CD14*, *LYN*, *SYK*, and *CXCL8* was higher in the CK fragments. *CD14* participates in TLR2 and TLR4 recognition [36]. *LYN* and *SYK* are involved in immune receptor signaling, whereas *CXCL8* promotes inflammatory cell migration and local inflammation [37,38]. ALA has been reported to reduce *CD14* recruitment to lipid rafts in macrophages [39]. ALA has also been reported to suppress NF-κB and MAPK signaling and reduce the production of proinflammatory factors [39,40]. The macrophage marker gene set was also enriched in the CK fragments. In orchitis models, activated testicular macrophages can induce germ cell apoptosis and inhibit spermatogonial proliferation through TNF-α and other inflammatory factors regulated by NF-κB [41,42]. TNF-α can also reduce occludin, ZO-1, and N-cadherin levels, disrupt the blood–testis barrier, and impair Sertoli–germ cell adhesion [43]. Together, these findings suggest that ALA treatment was associated with weaker immune-related transcriptional signatures in cultured testicular tissue fragments. However, these results do not demonstrate a direct effect of ALA on inflammatory signaling.

Exploratory transcriptomic analysis further showed that gene sets related to ATP-dependent chromatin remodeling, DNA replication, and male meiotic nuclear division were enriched in fragments treated with ALA. Before entering meiotic prophase, germ cells must complete DNA replication. Meiotic prophase also requires extensive chromatin remodeling and homologous recombination. Therefore, chromatin remodeling, DNA replication, and DNA repair are essential for the initiation and progression of meiosis [44]. Previous studies have shown that ALA increases the mRNA expression of *Cyclin B1* and *Bcl2* and promotes cell survival and proliferation [4]. ALA has also been reported to regulate enzymes involved in histone acetylation and DNA methylation [45]. In the present study, the expression of *TRRAP*, *POLE*, and *CHTF18* was higher in fragments treated with ALA. Conditional deletion of *TRRAP* in mammalian cells reduced histone H4 acetylation at DNA double-strand break sites and impaired homologous recombination repair [46]. *POLE* may contribute to the completion of recombination and DNA damage repair during the later stages of meiosis [47]. In mice, *Chtf18* knockout delayed the repair of meiotic DNA double-strand breaks and caused homologous chromosomes to separate earlier than normal [48]. These meiotic defects were accompanied by lower sperm counts and impaired spermatogenesis [48]. The secondary spermatocyte marker gene set was also enriched in fragments treated with ALA, whereas the A-dark spermatogonia marker gene set showed a trend toward enrichment. The higher expression of *SYCP3* and *MKI67* was consistent with the enrichment of gene sets related to cell proliferation and meiosis. Together, these findings suggest that ALA treatment was associated with stronger cell proliferation- and meiosis-related transcriptional signatures in cultured testicular tissue fragments. However, these results do not demonstrate a direct effect of ALA on DNA replication or meiotic progression.

This study has several limitations. The main limitation is that all testicular tissue fragments were obtained from a single ram. Although multiple fragments were cultured independently, they do not represent independent biological replicates at the animal level, which introduces a risk of pseudoreplication. Therefore, this study is an exploratory pilot study. In addition, the changes in ATP-dependent chromatin remodeling, DNA replication, and meiosis were mainly supported by RNA sequencing and cell-type marker gene set analysis. These changes may also be related to differences in cell composition or cell survival between the cultured fragments. Further studies are needed to validate these findings by measuring cell proliferation, meiotic marker proteins, and cell ploidy. ALA uptake and metabolism and changes in tissue lipid composition were also not measured. In future studies, tissues from multiple independent rams will be used to further confirm these findings.

## 5. Conclusions

In conclusion, fragments treated with ALA showed a greater seminiferous tubule diameter and a lower proportion of empty space on culture days 30 and 50, as well as more UCHL1-positive cells per seminiferous tubule on day 50. Exploratory transcriptomic and qRT-PCR analyses showed differences in gene expression related to inflammation, cell proliferation, and meiosis between the CK and ALA-treated fragments. These findings provide preliminary information on the response of cultured sheep testicular tissue fragments to ALA.

## Figures and Tables

**Table 1:** Primer sequence information.

Genes	Primer Sequence (5′–3′)	Product Size (bp)
*CHTF18*	F: GGACCTCCAAGGAACAGCAA	97
XM_042239764.2	R: GTTTGATGTGGGGAGTAGGGG	
*CXCL8*	F: CAACGGAAAAGAGGTGTGCTT	89
NM_001009401.2	R: GATCTTGCTTCTCAGCTCTCTTCA	
*LYN*	F: ACTCACACACGGAGCTGAATG	76
XM_060419898.1	R: TTCTCTCCCTTCACATCGGC	
*NFKBIA*	F: TCACCTACCAGGGCTACTCC	178
NM_001166184.1	R: CAGTCATCGTAGGGAAGCTCA	
*POLE*	F: TCACCATCCACACTAAGGTCTTCA	169
XM_042234450.1	R: GGACACAGGAGCAGAGGACA	
*TRRAP*	F: GGAGTGACGGAAATGAGAGCA	93
XM_060405713.1	R: TCTCCTCTACCCCTTCCACG	
*SYCP3*	F: GAGTATGGAGGACTTGGAGAAGA	122
XM_004006668.5	R: CCATCTCTTGCTGCTGAGTTTC	
*MKI67*	F: GATGTCTGACCTTCCCGTCG	153
XM_042238739.2	R: CACTCGACACCCCTTCCAAA	
*ZBTB16*	F: ATTCGGCTTGTTCCCTTCCTG	142
XM_027979222.3	R: ATGGCAAGGGCCCGACTG	
*HPRT1*	F: CGACTGGCTCGAGATGTGAT	197
XM_015105023.2	R: TCACCTGTTGACTGGTCGTT	

## Data Availability

The raw RNA-seq data generated in this study have been deposited in the CNGB Sequence Archive (CNSA) of the China National GeneBank DataBase (CNGBdb) under accession number CNP0009998.

## Supplementary Materials

The following supporting information can be downloaded at https://www.mdpi.com/article/10.3390/ani16182873/s1, Supplementary Methods: Complete culture medium formulation. Table S1: Summary of RNA-seq data quality. Table S2: Differentially expressed genes between the ALA and CK groups in cultured sheep testicular tissue. Table S3: Complete GO and KEGG gene set enrichment analysis results for the ALA and CK groups. Table S4: Cell type marker gene set enrichment analysis results for the ALA and CK groups across three marker selection strategies. Table S5: Gene level count matrix.

## Abbreviations

The following abbreviations are used in this manuscript:

ALA	α-linolenic acid
CK	Control
DEGs	Differentially expressed genes
FDR	False discovery rate
GO	Gene Ontology
GSEA	Gene set enrichment analysis
KEGG	Kyoto Encyclopedia of Genes and Genomes
log_2_FC	Log_2_ fold change
NES	Normalized enrichment score
T-AOC	Total antioxidant capacity
