# Supplementary material for: Effects of α-Linolenic Acid on Seminiferous Tubule Morphology and UCHL1-Positive Cells in Cultured Testicular Tissue Fragments from a 3-Month-Old Hu Ram: A Pilot Study
Source: Animals (Basel). 2026 Sep 12;16(18):2873. doi: 10.3390/ani16182873 (PMC13603160; doi:10.3390/ani16182873)
Supplement: Supplementary file 1 [file animals-16-02873-s001.zip › Supplementary Material.pdf]

Supplementary Methods: Complete culture medium formulation

Complete medium for sheep testicular tissue fragment culture was prepared using KnockOut™ DMEM (Gibco, Waltham, MA, USA, 10829018) as the basal medium. The medium was supplemented with 10% KnockOut™ Serum Replacement (Gibco, Waltham, MA, USA, 10828028), 10 ng/mL glial cell line derived neurotrophic factor (Gibco, Waltham, MA, USA, PHC7044), 5 IU/L follicle stimulating hormone (Sigma Aldrich, St. Louis, MO, USA, 869001-M), 1 IU/L chorionic gonadotropin (Ningbo Second Hormone Factory, Ningbo, China), 1 μM retinol (Sigma Aldrich, St. Louis, MO, USA, R7632), 34 nM α-tocopherol (Sigma Aldrich, St. Louis, MO, USA, 258024), 1% MEM nonessential amino acids (Gibco, Waltham, MA, USA, 11140050), 2 mM L-glutamine (Sigma Aldrich, St. Louis, MO, USA, G6392), 1 mM sodium pyruvate (Sigma Aldrich, St. Louis, MO, USA, P4562), 1% penicillin-streptomycin (Gibco, Waltham, MA, USA, 15140122), 1 μM testosterone (Aladdin, Shanghai, China, T102170), 100 nM 17β-estradiol (Aladdin, Shanghai, China, E110145), 0.05 mg/mL L-ascorbic acid (Sigma Aldrich, St. Louis, MO, USA, A4403), 20 ng/mL human epidermal growth factor (Gibco, Waltham, MA, USA, PHG0311), 20 ng/mL human basic fibroblast growth factor (Gibco, Waltham, MA, USA, PHG0264), 10^{-7} M melatonin (Thermo Scientific Chemicals, Waltham, MA, USA, J62452.06), 3 IU/L pituitary luteinizing hormone (Ningbo Second Hormone Factory, Ningbo, China), 2 ng/mL 3,3′,5-triiodo-L-thyronine sodium salt (Thermo Scientific Chemicals, Waltham, MA, USA, J63312), 1 mM N-acetyl-L-cysteine (Thermo Scientific Chemicals, Waltham, MA, USA, A15409.14), and 100 μM reduced L-glutathione (Thermo Scientific Chemicals, Waltham, MA, USA, J62166.06). After all components were added, the medium was passed through a sterile 0.22 μm membrane filter and warmed to 37 °C before tissue culture.
